# Hold up Our Hands

**Published:** 1885-07

**Authors:** 


### HOLD UP OUR HANDS.

We cordially and most earnestly call on the physicians of Texas to aid us in making of this undertaking a grand success. Reflect what credit journalism has already brought to the profession of Texas; how organization has been promoted; how medical matters have taken on a new impulse; how the profession has gained additional prominence in the National Association, and chiefly through the aid voluntarily given a Texas journal by a few. If the older and better known practitioners will but contribute each month, a mite of their experience, observation and reflection, in short articles, to the pages of this Journal, they will render a signal service to the profession at large, and to us individually, who are working in the good cause, *con amore*. If the profession *will but hold up our hands*, the battle will be won, and Texas will have a journal which will be an honor to the State.

We need original matter *now*, for next issue, and *all* the time. *Write now*.

